# Sensory fusion in the hoverfly righting reflex

**DOI:** 10.1038/s41598-023-33302-z

**Published:** 2023-04-15

**Authors:** Anna Verbe, Dominique Martinez, Stéphane Viollet

**Affiliations:** 1 Aix-Marseille Université, CNRS, ISM, 13009 Marseille, France; 2 Université de Lorraine, CNRS, LORIA, 54000 Nancy, France; 3 PNI, Princeton University, Washington Road, Princeton, NJ 08540 USA

**Keywords:** Neuroscience, Computational neuroscience, Sensory processing

## Abstract

We study how falling hoverflies use sensory cues to trigger appropriate roll righting behavior. Before being released in a free fall, flies were placed upside-down with their legs contacting the substrate. The prior leg proprioceptive information about their initial orientation sufficed for the flies to right themselves properly. However, flies also use visual and antennal cues to recover faster and disambiguate sensory conflicts. Surprisingly, in one of the experimental conditions tested, hoverflies flew upside-down while still actively flapping their wings. In all the other conditions, flies were able to right themselves using two roll dynamics: fast ($\sim$50ms) and slow ($\sim$110ms) in the presence of consistent and conflicting cues, respectively. These findings suggest that a nonlinear sensory integration of the three types of sensory cues occurred. A ring attractor model was developed and discussed to account for this cue integration process.

## Introduction

In the righting reflex, animals have to reorient themselves to reach an upright position. This is an example of goal-directed behavior^1,2^ in which a goal signal (in this case, the right-side up orientation) is compared with an estimate of the current state (the body roll movement). The animal eventually reaches the goal position by canceling any closed-loop errors (i.e., any differences between targeted and current states). In the framework of control theory, many kinds of behavior can be said to function like feedback control systems on the basis of responses to disturbances or perturbations^3–7^. Electromagnetic pulses applied to a metallic pin attached to a fruitfly’s body have shown, for example, that these insects are able to completely reject disturbances in their roll^8^, pitch^9^, and yaw movements^10^ within a few milliseconds. Feedback control systems provide insects with a highly efficient means of ensuring stable locomotion, compensating for morphological variability^11^ and even rejecting perturbations, for example, in order to maintain the appropriate heading^12^. Goal-directed behavior raises the question, however, as to how internal goal signals are generated on the basis of sensory cues.

We addressed this question by investigating the multisensory integration process at work in the hoverfly righting reflex. When falling upside-down, hoverflies trigger their wingbeats in order to rotate and regain the right-side up position within a short lapse of time (mean value: 48.8ms, see^2^). During the righting, hoverflies may produce the goal roll via three main sensory pathways: visual, leg proprioceptive, and antennal airflow sensing. The dorsal light response (DLR), a visual reflex, enables flies to determine their orientation, since the brightest part of the environment is presumably located above them^13–17^. Leg proprioceptive cues might also be used via the tarsal reflex to measure the angle of the surface on which flies are standing^13–18^. The fly’s antennae may also play the role of mechanoreceptors during flight^19–21^ by detecting the direction of the airflow and its changes^22–24^.

Our working hypothesis was therefore that the goal roll signal triggered during the righting reflex is based on the sensory integration of antennal, visual, and leg proprioceptive cues. Our experiments combined with computational modeling show that hoverflies integrate antennal, visual, and leg proprioceptive cues and trigger a goal roll signal. Interestingly, the righting was two times slower when the sensory cues disagreed, or in the experiments performed in the dark. One particular sensory conflict even led to an unexpected stable inverted flight with no righting. These results suggest that a nonlinear sensory integration occurs in the hoverfly righting reflex. We further developed a ring attractor model accounting for these nonlinear dynamics. A ring attractor network is a biologically plausible neural network underlying sensory cue integration^25^. It can be useful to combine conflicting cues of various strengths^26^ and can even perform Bayesian inference^27^. Our model is based on a ring attractor network with a global inhibitory neuron^28,29^ extended with sigma-pi neurons accounting for the nonlinear response of the hoverfly.

## Results

### The roll righting reflex has a multi-sensory basis

**Figure 1:**
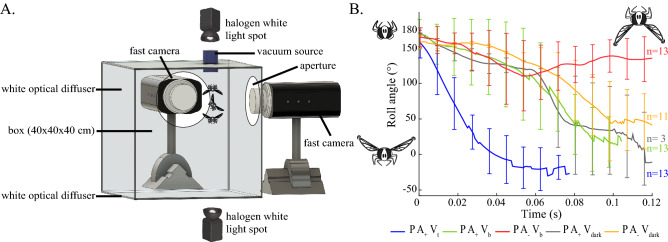
Experiments (**A**) The experimental set-up used here to analyze the hoverfly aerial righting reflex. The observation cage consisted of a transparent $40\times 40\times 40$ cm PVC box, all the sides of which were covered with a white diffuser. Illumination was provided from above or below by a halogen light. The fly was held upside-down on the ceiling of this box using a vacuum set-up^2^. When the vacuum source was turned off, the fly was released and started falling. The fall was recorded with two high-speed video cameras facing two adjacent sides of the box at a rate of 1690 fps in full resolution ($1280\times 800$ pixels). (**B**) Plot of the body roll angle versus time ($\theta _{TR}$) in the following five conditions: $P A_+ V_t$ (in blue, with the antennae intact and lit from the top), $P A_+ V_b$ (in green, with the antennae intact and lit from below), $P A_+ V_{dark}$ (in grey, with the antennae intact and placed in the dark), $P A_- V_b$ (in red, with the antennae blocked and lit from below), $P A_- V_{dark}$ (in orange, with the antennae blocked and placed in the dark). Right-side up and upside-down orientations correspond to roll angles of 0$^\circ$ and 180$^\circ$, respectively. We did not observe any noticeable righting maneuvers involving body pitch or yaw rotations (Supplementary Movies S1 to S5). Thick lines are means and error bars are SDs.

Flies were placed in a free fall situation by releasing them upside-down from a suction-based custom-built device^2^ (Fig. 1A). Their body roll orientation was analyzed closely versus time, using two fast cameras (Supplementary Table S1). Flies were exposed to various sensory conditions during the free fall (Supplementary Movies S1 to S5). Their righting reflex was expected to depend on the integration of three sensory inputs: leg proprioceptive cues (*P*), dorsal light responses involving vision (*V*), and airflow cues involving the antennae (*A*). The flies had prior knowledge of their orientation with respect to gravity, as they were always in contact with the ceiling before being released (*P*). As a fly deprived of proprioception before being released crashes irremediably onto the ground^16,30^, we did not include any condition without proprioception. Sensory conflicts were introduced by varying the visual inputs (lighting from above or below) and the state of the antennae (glued or intact). The various combinations of stimuli used either triggered righting behavior or not. As shown in Figure 1B, the following five conditions based on sensory cues were therefore tested: with proprioception *P*, with the antennae *A* either intact ($A_+$) or glued ($A_-$) and under three visual conditions *V* (with lighting from above $V_t$, or below $V_b$ or in the dark $V_{dark}$).

### Depending on the sensory cues available flies performed either proper righting maneuvers or inverted flight

Proper righting occurred in the three visual conditions involving intact antennae (Fig. 1B) with the setup lit from above ($P A_+ V_t$, Supplementary Movie S1), below ($P A_+ V_b$, Supplementary Movie S3) and in the dark ($P A_+ V_{dark}$, Supplementary Movie S4). Flies were able to right themselves in the condition $P A_- V_{dark}$, which means that the presence of proprioceptive cues alone sufficed to trigger the righting reflex. However, Campaniform sensilla located along the body and legs might also contribute, but this hypothesis was not tested here. Complete righting was always observed with intact antennae ($A_+$), regardless of the visual conditions. When the condition $A_-$ was combined with either one of the two visual conditions ($V_b$ or $V_{dark}$), opposite responses were observed: either complete righting ($P A_-V_{dark}$, Supplementary Movie S5) or sustained inverted flight ($P A_-V_b$, Supplementary Movie S2).

As a stable inverted flight was observed in the condition $P A_- V_b$, we analyzed the transient roll dynamics leading the fly to trigger wingbeats while maintaining an upside-down position. In this condition, the righting process started normally as in the three conditions $P A_+ V_b$, $P A_+ V_{dark}$ and $P A_- V_{dark}$, but was reversed at 90.79±46.80$^\circ$ and 60.39±27.60ms (means ±SDs, red curve in Fig. 1B). A final roll orientation of 158.2±20.89$^\circ$ (mean ±SD) with respect to the initial upside-down position was reached within 99.53±46.16ms (mean ±SD, Supplementary Movie S6).

### Depending on the sensory cues available flies performed either fast or slow righting reflex

Apart from the condition $P A_- V_b$, proper righting of the fly was consistently observed. We then examined whether various sensory combinations had any effect on the righting reflex. In the condition $P A_+ V_t$, flies righted themselves within 48.8±10.9ms (mean ±SD, blue curve in Fig. 1B) of the first wingbeat. In addition, their roll responses featured a fast transient phase followed by a steady-state phase corresponding to a body roll of 0$^\circ$ (right-side up). In the condition $P A_+ V_b$, flies righted themselves within a longer time of 83.03±17.58ms (mean ±SD, green curve in Fig. 1B). These results are similar to those previously reported in^2^. In the condition $P A_+ V_{dark}$, flies also righted themselves, but within 107.4±29.39ms (mean ±SD), which is 58.64 ms longer than in the condition $P A_+ V_t$ (mean, gray curve in Fig. 1B). In the dark without the use of their antennae ($P A_- V_{dark}$), flies took 142.8±38.7ms to right themselves (mean ±SD, yellow curve in Fig. 1B). To summarize, in the presence of a sensory conflict or in the absence of some sensory cues, the righting behavior was always slower than in the condition $P A_+ V_t$ ($\sim$110ms vs $\sim$50ms).

### A ring attractor model accounts for the slow/fast righting dynamics

**Figure 2:**
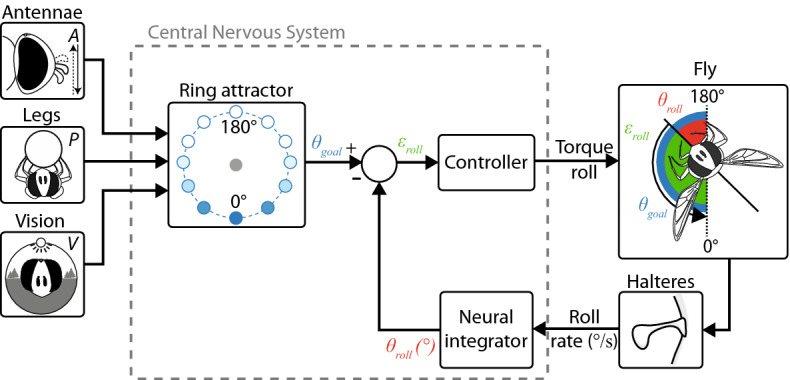
Tentative model for the roll righting reflex in flies. By merging the three parallel sensory pathways (the antennal, leg proprioceptive and visual pathways), the ring attractor delivers a goal roll signal $\theta _{goal}$ to the closed-loop controlling the insects’ body roll movements. The controller drives the fly’s body roll $\theta _{roll}$ so as to follow $\theta _{goal}$ by canceling the closed-loop error $\epsilon _{roll}$. The $\theta _{roll}$ signal results from the integration of the roll rate sensed by the fly’s halteres.

**Figure 3:**
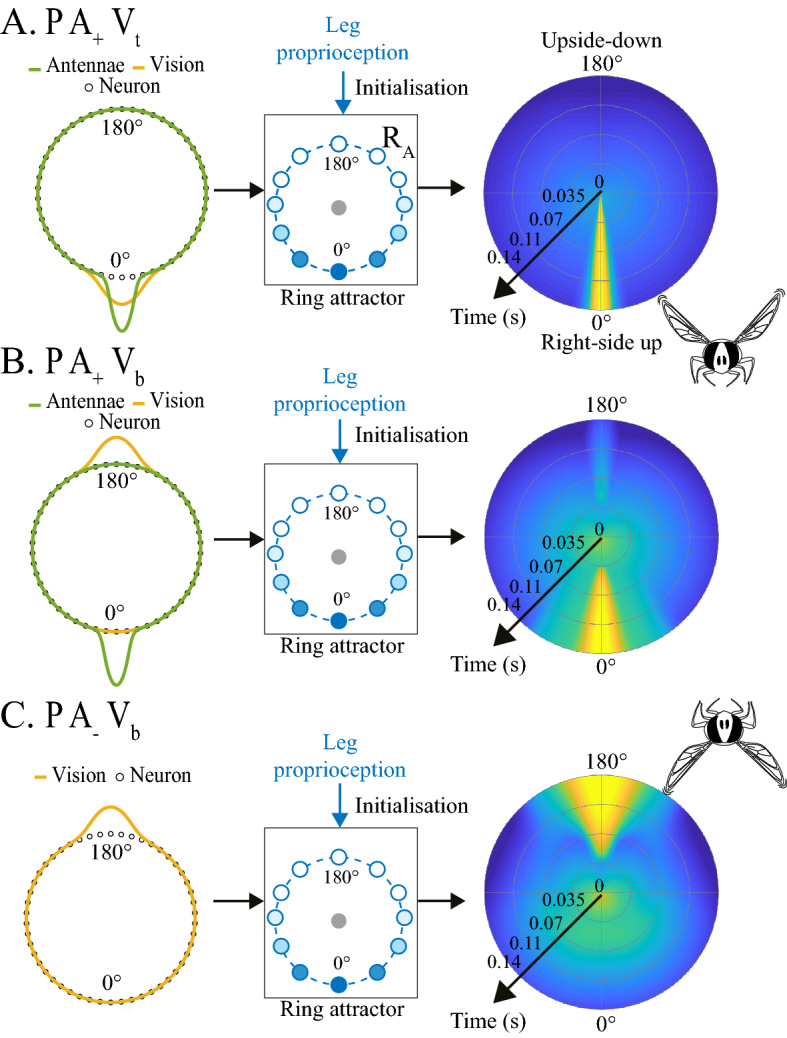
Ring attractor responses in the three conditions $P A_+ V_t$, $P A_+ V_b$ and $P A_- V_b$. The output from the ring with time is expressed as the winner-take-all (winner angle) function, that is, at each time step, the winning neuron is that featuring the highest level of activation. The winner angle codes for the roll state value targeted $\theta _{goal}$ (Fig. 2), whereas the K value is equal to the amplitude of the winning neuron’s activation (Supplementary Fig. S2). The initialization of the ring attractor is affected by the leg proprioceptive signal in the three conditions and the integration of the orientation cues (antennal and visual). In the case of $P A_+ V_t$, the antennal and visual cues both give the same information corresponding to an upside-down position (0$^\circ$), while in the condition $P A_+ V_b$, the information conveyed by the antennal cues corresponds to an upside-down position (0$^\circ$) and that conveyed by the visual cues corresponds to a right side up position (180$^\circ$). In the last condition tested, $P A_- V_b$, the antennae were blocked, which meant that only the visual cues were integrated resulting in a right-side-up position (180$^\circ$). Initialization at $t=0$ is the same in all three conditions: K = 0.45, Winner angle = 0$^\circ$. The final state at $t=0.14$ s was K = 178, Winner angle = 0$^\circ$ (panel **A**), K=3.21, Winner angle = 0$^\circ$ (panel **B**) and K=1.64, Winner angle = 180$^\circ$ (panel **C**). Color code in right column is defined as follows: blue=low activation, green=medium activation, yellow=high activation.

The tentative model developed here (Fig. 2, see details in Supplementary Sect. 1.1) integrates sensory information accounting for the various righting speeds (fast and slow) observed. Each neuron in the ring had a preferred orientation. A rotational symmetry was assumed to exist around the ring so that the preferred orientations were evenly distributed around (0$^\circ$, 360$^\circ$), with the same neuron encoding for 0$^\circ$ and 360$^\circ$. Simulations were performed with 100 neurons so as to ensure a sufficiently high level of angular precision, but the results obtained with this model are robust to the choice of ring attractor size. The ring attractor was initialized with a wide Gaussian bump at 0$^\circ$, corresponding to the proprioceptive cues (*P*) sensed by the insect’s legs prior to the fall. The other two sensory pathways were simulated in the form of Gaussian inputs, $X_A$ and $X_V$ (antennae and vision), to the ring attractor (see Fig. 3). Both vision and leg proprioception were established before the free fall. However, we assumed that leg proprioception initialized the ring attractor as this was the only cue no longer available during the free fall. This assumption requires further experiments in order to understand the role of leg proprioception and vision prior to the free fall more clearly.

We first dealt with the linear neurons in the ring, to which the total input was the sum of the two sensory inputs $X_A+X_V$. With linear neurons, however, we did not completely succeed in modeling the change in dynamics between consistent and conflicting cues (Supplementary Fig. S6B). We, therefore, used high-order sigma-pi units^31,32^ to which the total input was computed by taking the weighted sum of the product of two individual inputs $X_A + X_V + \omega \; X_A \;X_V$, where $\omega$ is a weighting factor. As a result, when the two sensory cues are in conflict, the Gaussians are far from each other (e.g. with $X_A$ and $X_V$ centered at $0^\circ$ and $180^\circ$) and the product $X_A \;X_V \approx 0$. The sigma-pi unit then behaves like a linear neuron with the input $X_A + X_V$. By contrast, when the two sensory cues are in agreement, the Gaussians are close to each other and the product $X_A \;X_V$ is large, resulting in a nonlinear amplification of the input.

The output of the ring attractor over time (Fig. 3 and Supplementary Fig. S4) is encoded as the winner-take-all solution, that is, at each time step, the winning neuron is that showing the highest level of activation (Supplementary Figs. S7 and S8). The goal roll signal is obtained by filtering the winner angle with a time constant $\tau _f \propto 1/K$, where *K* is the amplitude of the winner activation (filter implementation in Supplementary Figs. S3 and S8). The role of the filter is to introduce some dynamics in the goal roll signal so that a weakly activated winner would produce the slow dynamics observed in the presence of sensory conflicts. The roll control was modeled in the form of a closed-loop system (Fig. 2): The goal roll signal $\theta _{goal}$ is compared with the fly’s body roll $\theta _{roll}$ estimated from the roll rate sensed by the halteres. A complete block diagram of the fly’s roll feedback control system is presented in Supplementary Fig. S2.

**Figure 4:**
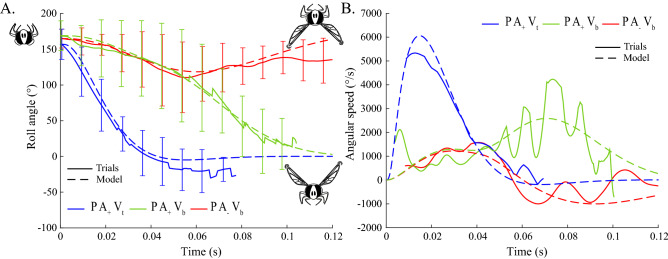
Model versus Experiments. Response of the righting reflex model (dotted line) versus the experimental data (solid line) in terms of the roll angle (**A**) and the angular speed (**B**) in three of the main conditions tested: with the antennae intact, lit from above, $P A_+ V_t$ in blue, and from below, $P A_+ V_b$ in green. With the antennae glued, lit from below, $P A_- V_b$ in red. Thick lines are means and error bars are SDs. See Supplementary Sect. 1.1.

Figure 4A and Supplementary Movies S1 to S3 show the responses of the model versus the roll responses of the fly in the three experimental conditions $P A_+ V_t$, $P A_+ V_b$, and $P A_- V_b$. The model was also tested in the two conditions in the dark $P A_+ V_{dark}$ and $P A_- V_{dark}$ (Supplementary Figs. S4 and S5, and Movies S4 to S5). The goal roll signal $\theta _{goal}$ featured a fast transient when the sensory cues were consistent and a slower transient when the sensory cues were in conflict or in the dark. The results of the simulations, therefore, matched the fast and slow dynamics observed in the fly’s body roll. In addition, the simulated response corresponding to the condition $P A_- V_b$ featured a similar back-and-forth rolling maneuver. In line with the experimental findings, the simulated roll righting stopped suddenly 60ms after its onset, at a roll of $118^\circ$, before rotating back to a similar upside-down position to its initial position($164^\circ$).

The fastest righting response (50ms) was observed and simulated in the condition $P A_+ V_t$. It took only 15ms for the simulated roll rate to reach a maximum speed of 6061$^\circ$/s, which is similar to that observed in the experiments (6490±1111 $^\circ$/s at 15.14±5.73ms, means ±SDs, Fig. 4B). Slower righting responses were also obtained in the simulations in the three conditions $P A_+ V_b$ (Fig. 4B), $P A_+ V_{dark}$ and $P A_- V_{dark}$ (Supplementary Fig. S5B). In these three conditions, the righting lasted for about $\sim$100ms with an angular speed of around 944$^\circ$/s (mean value recorded during the first 50ms, which was similar to the slower roll rate observed experimentally in the hoverfly, see Fig. 4B). In both experiments and simulation, an increase in the roll angular speed was also observed after 50ms in the two conditions $P A_+ V_b$ (Fig. 4B, green curve) and $P A_+ V_{dark}$ (Supplementary Fig. S5B, grey curve).

## Discussion and conclusion

### A closed-loop roll control based on the halteres

To control its body roll during the righting process, the fly has to estimate the current roll on the time scale of a single wingbeat. In this context, vision is probably not fast enough. Hoverfly righting is entirely achieved within 6 wingbeats, i.e., 50ms^2^, and the processing time of the visual system in hoverflies is probably of the order of that measured in blowfly *Calliphora* ( ± 20*ms*)^33,34^, even if this latency depends strongly on the experimental conditions (lighting intensity, contrast amplitude, temperature...). Therefore, roll control on the timescale of a single wingbeat is not compatible with visual processing, whereas the halteres are fast sensors for measuring the roll rate^10,35^. It was therefore assumed that the body roll is estimated by integrating the roll rate given by the halteres (Fig. 2 and Supplementary Fig. S2). In line with the results obtained with our model, it is worth noting that modifying the halteres, e.g. by adding mass, drastically affected the flight dynamics^2,36^. Neither vision nor antennae are required for body roll stabilization to occur, as hoverflies with blocked antennae placed in the dark are able to right themselves correctly ($P A_- V_{dark}$, Fig. 1 and Supplementary Movie S5)).

### A goal roll signal based on three types of external cues

Contrary to what was reported in a previous study suggesting that vision is necessary to the righting reflex in dragonflies^37^, complete righting of hoverflies was observed here in the dark ($P A_{+} V_{dark}$, $P A_{-} V_{dark}$). This discrepancy between dragonflies’ and flies’ performances may be attributable to the differences between the initial conditions prior to the free fall, i.e., no leg proprioception was present in Wang et al, 2022^37^ whereas leg proprioception was present via the substrate (*P*) in the present study. We previously reported that a fly deprived of leg proprioception before being released in the dark crashes irremediably onto the ground^30^, but it has emerged that flies right themselves correctly when their legs are in contact with a substrate before being released. In the present experimental setup, there were no moving parts or possible leg kicks that might have introduced any marked variability in the initial conditions. As shown in the movies (Supplementary Movies S1 to S5), our device based on a vacuum source^2^ always gave the flies a smooth take-off. Leg proprioceptive cues may therefore have provided sufficient prior knowledge of the initial roll orientation for the flies to be able to recover their position during the free fall.

To support this idea, upon running our ring attractor with leg proprioceptive cues alone ($P A_{-} V_{dark}$, Supplementary Figs. S4 and S5), we noted that these cues sufficed to generate an effective internal goal roll signal driving the righting sequence. Leg chordotonal organs are known to sense the changes in load which occur with respect to gravity and the distribution of weight relative to the substrate^38–40^. In addition, in the fruitfly, three kinds of mechanosensory neurons have been found to code for the angular rate and the orientation of the leg joints^41^. Secondly, contrary to the prevailing belief that the halteres are involved in gravity perception^42,43^, the possible involvement of these organs to generate the goal roll signal $\theta _{goal}$ was unlikely in this study, as no righting (but inverted flight) was observed in the condition $P A_- V_b$ where the fly triggered the wingbeat and thus the halteres’ vibration. Note that in this condition, in which the antennae were glued, the inverted flapping flight was stable, unlike the unstable flight observed in hawkmoths with clipped-off antennae^44,45^. This discrepancy is probably due to the fact that Dipterans, unlike hawkmoths, have halteres which contribute greatly to stabilizing the insects’ flight by acting like rate gyros^35,46,47^. The fact that different behavior was observed here in the condition $P A_+ V_b$ vs $P A_- V_b$ suggests that antennae are involved in producing the goal roll signal with respect to either the airflow direction or gravity. Antennal mechanosensors have been found to contribute to sensing the airflow^48–50^ and maintaining the headwind orientation^51^. Another recent study on Drosophila has shown that wind-induced antennal displacements affect the compass of the fly’s brain^23^. In insects, antennal mechanosensory mediation takes place in the Johnston’s organ, which is a highly sensitive mechanosensory structure located in the antennal pedicel-flagellar joint^22,52^. However, the possible involvement of the hoverfly’s antennae in gravity perception requires further experiments to assess its role in the absence of leg proprioception and vision. The stable inverted flight ($P A_- V_b$) and the difference in the roll dynamics observed in condition $P A_+ V_b$ vs $P A_+ V_t$ provide additional evidence that visual cues mediated via the DLR play a key role in measuring the fly’s absolute body orientation^16^.

### Linear versus nonlinear cue integration

How may leg proprioceptive, visual, and antennal cues be integrated to provide a reliable goal roll signal? Let us assume that the antennae and vision yield individual noisy goals with means $\mu _A$, $\mu _V$, and variances of $\sigma _A^2$ and $\sigma _V^2$; respectively (Supplementary Sect. 1.1). According to the minimum variance estimation rule^53^, the appropriate roll movement is given by a linear combination of the means weighted by their inverse variances; that is, $\omega \; \mu _A + (1-\omega ) \; \mu _V$, where $\omega = \sigma _A^{-2}/(\sigma _A^{-2}+\sigma _V^{-2})$. Applying this formula to the condition $P A_+ V_b$ gives $36^\circ$ so that the fly can be expected to turn only partially. However, this situation was not observed in the present experiments as the flies always performed either right-side up ($0^\circ$) or upside-down ($180^\circ$) rotations. We nevertheless simulated a linear cue integration model and show that this cannot account for the experimental data (Supplementary Fig. S6). These results, therefore, suggest that flies do not perform linear cue integration as stated by the minimum variance estimation rule.

In addition, the nonlinear nature of the righting is confirmed by the differences in response time, depending on whether the sensory cues are in conflict or in agreement. This result is in contradiction with linear cue integration models on hawkmoths^54^ and humans^55^ based on experiments involving small amplitude variations in sensory conflicts. However, nonlinear sensory cue integration has been found to occur in previous studies^56,57^. In addition, since the sensory conflicts ($A_{+}$ vs $A_{-}$, $V_{t}$ vs $V_{b}$) and roll amplitudes (0-$180^\circ$) were too large in the present experiments, linear modeling approximation is not applicable here. We, therefore, modeled the righting reflex in the form of a closed-loop control system in which the goal roll angle is provided by a ring attractor network (Fig. 2). The present model performs nonlinear cue selection in the presence of large sensory conflicts, where the strongest cue predominates over the others^26^. It also includes internal dynamics accounting for the slow versus fast righting responses observed in the experiments. When the sensory cues available are in conflict, the sigma-pi units^31,32^ of the ring attractor model behave like linear neurons, resulting in a small winner activation and a slow righting process. When the sensory cues are in agreement, the multiplication of the inputs induced by the sigma-pi units generates a large winner activation and a fast righting (Fig. 4). Here, the multiplication of sensory cues rather than linear integration accounts for the change in dynamics. Yet, our ring attractor model remains tentative in regards to the lack of knowledge in the neuronal processing of hoverflies and we cannot rule out the possibility that other nonlinear models might explain the data equally well.

### Biological plausibility of the ring attractor model

In line with Touretzky’s network^28^ (Supplementary Fig. S1), which was simulated with large sensory conflicts^26^, the present ring attractor model acts like a winner-take-all network by selecting the strongest cue. The main difference here in comparison with the previous ring attractor models on rodents^58^ and fruitflies^59^ is the use of sigma-pi neurons accounting for the nonlinear response (much faster response with consistent cues). It is worth noting that there exists evidence that individual neurons in the fruitfly can perform multiplications of their inputs^60,61^. The results obtained in this study correlate well with the key features of ring attractors^62^, namely: their responsiveness to the position of external stimuli, the persistence in the absence of external stimuli ($P A_+ V_{dark}$ and $P A_- V_{dark}$), locking onto a single external stimulus when presented with two competitors ($P A_+ V_b$ and $P A_- V_b$) and sliding between positions ($P A_- V_b$). What might the neural basis of the ring attractor model be in the fly brain? The central complex (CX) in insects is a key brain area involved in the performance of spatial orientation and navigation tasks. The CX neural circuit’s activity has been found to track the insect’s current heading relative to its environment. Recent studies have shown that part of the CX network can be modeled in the form of a ring attractor^63^. In addition, it has been established that in various insect species (^64^ for a review), many neurons in the central complex integrate multisensory information by responding to various (mechanical, visual, and olfactory) stimuli. However, even if the layout of the CX is conserved among species, the idea that the CX is the substrate of nonlinear multisensory fusion cannot be generalized because it depends on whether the stimuli involved generate nonlinear responses, i.e., large sensory conflicts. The fact that the initial bump of activity due to the onset of leg proprioception (*P*) in our ring attractor model is present even in the absence of sensory inputs during the righting response ($P A_- V_{dark}$, Supplementary Fig. S5) suggests that the ring attractor may play the role of a working memory. In line with the model presented here, there exists experimental evidence that ring attractor networks maintain persistent activity for several seconds in *Drosophila*^59^.

## Conclusion

Voluntary movements in animals depend on their ability to generate internal goal signals controlling the value of current states. The present study focuses on the generation of the goal signal used to control the hoverfly’s roll in closed-loop. Here we have presented a ring-attractor model of how this signal may be obtained based on a nonlinear multisensory integration. We have stressed the key role played by sensory redundancy in the righting reflex, as flies can experience various conflicting sensory situations in real life, e.g., when the ground is not always darker than the sky or the antennae are affected mechanically by dust or pollen. The ring attractor mechanism present in the brain of vertebrates and invertebrates has been shown to carry an estimate of the current heading. However, the neural substrate of a goal roll signal has not been identified so far. The findings in this study shed a new light on the role of ring attractors in the robust coding of goal orientation.

## Methods

### Biological material

*Episyrphus balteatus* pupae were purchased from Katz Biotech AG, Baruth, Germany. They were fed *ad libitum* with pollen and honey. Flies were released using a custom-built suction-based device, as in a previous study (See Verbe et al (2020)^2^ for further information). The following changes were made to the previous set-up: a white optical diffuser was added to the sides of the box and two apertures were made on adjacent sides in order to be able to film the falling flies with two fast cameras (Fig. 1A).

### Experimental set-up and procedure

In the conditions $P A_- V_b$, the flies’ aerial righting performances were recorded with two high-speed video cameras (Phantom VEO E310 and Phantom Miro M11O) at a rate of 1690 frames per second (resolution: 1280x800 pixels). The two cameras were positioned at an angle of 90$^\circ$ pointing towards the box in order to obtain 2D and 3D views of part of the fall. The two lenses used (a Nikon Micro-Nikkor AF-S DX Micro 40mm f/2.8 G and a Nikon Micro-Nikkor AF-S N 60mm f/2,8) gave a good compromise between the size of the fly, the resolution, and the visual field (the fly-to-camera distance was $\sim$ 20cm).

In the other four conditions ($P A_+ V_t$, $P A_+ V_b$, $P A_+ V_{dark}$ and $P A_- V_{dark}$), the same experimental set-up was used as that described in Verbe et al., 2020^2^ (Supplementary Table S1). In the condition with antennae blocked, a small drop of glue (fifty percent of rosin and bee wax) was deposited at the basis of each antenna so as to block any deflection due to the airflow. The experimental arena was covered at the top and on the sides with white diffusers (PMMA WH02, 3mm thick) and illuminated from above and below by a halogen light (Kaiser Studiolight H = $5.6 * 10^{-13} W.m^{2}$ and $1.76 * 10^{-11} W.m^{2}$, respectively). When the experimental arena was placed in the dark, infrared light projectors (BLANKO, wavelength 850 nm) were used to film the flies’ behavior. The VEO camera was triggered automatically as soon as the insect entered the camera’s field of view, which started the miro camera. To synchronize the two cameras exactly, infrared LEDs placed in both cameras’ fields of view were switched on automatically whenever the VEO camera was triggered.

In conditions $P A_+ V_t$, $P A_+ V_b$, $P A_+ V_{dark}$, $P A_- V_b$ and $P A_- V_{dark}$, a total number of 13 drops (4 males and 6 females), 13 drops (3 males and 1 female), 3 drops (1 male and 2 females), 13 drops (4 males and 3 females) and 11 drops (5 males and 3 females) were recorded. Note that at each drop, each fly experienced a single experimental condition.

#### Image processing and analysis

The same method as that described in^2^ with a Tracker Video Analysis and Modeling Tool was used here (Copyright (c) 2018 Douglas Brown). The other analyses were performed with MATLAB (R2018a, MathWorks, Natick, MA, USA).

### Ethics

No ethical authorization for animal research or permission to carry out fieldwork was required for this study.

## Supplementary Information

Supplementary Information 1.

Supplementary Information 2.

Supplementary Information 3.

Supplementary Information 4.

Supplementary Information 5.

Supplementary Information 6.

Supplementary Information 7.

## Data Availability

Source code of the ring-attractor model as well as data from this study have been deposited in GitHub (https://github.com/AnnaVerbe/Sensory_ring).
