# Supplementary material for: Sensory fusion in the hoverfly righting reflex
Source: Sci Rep. 2023 Apr 15;13:6138. doi: 10.1038/s41598-023-33302-z (PMC10105705; doi:10.1038/s41598-023-33302-z)
Supplement: Supplementary file 7 — Supplementary Information 7. [file 41598_2023_33302_MOESM7_ESM.pdf]

Supplementary document - Sensory fusion in the hoverfly righting reflex

Anna Verbe[a,c], Dominique Martinez[a,b], and Stéphane Viollet[a,*]

[a]Aix-Marseille Université, CNRS, ISM, 13009, Marseille, France.
[b]Université de Lorraine, CNRS, LORIA, 54000 Nancy, France.
[c]PNI, Princeton University, Washington Road, Princeton, NJ 08540, USA.
[*]corresponding: stephane.viollet@univ-amu.fr

1 Material and methods

Supplementary Table S 1. Camera configurations depending on the condition tested.

Experiment	Number of cameras	Frames per second	Linked article
PA_+V_t	1	1600	[1]
PA_+V_b	1	3200	[1]
PA_-V_b	2	1690	/
PA_+V_{dark}	1	3200	/
PA_-V_{dark}	1	1690	/

1.1 Ring attractor network

Here we describe the ring attractor model based on the experimental data. Each neuron i in the ring had a preferred orientation θ_i, $i = 1 \cdots n$. Rotational symmetry was assumed to exist around the ring so that the preferred orientations were evenly distributed around (0°, 360°), with the same neuron encoding for 0 and 360°. Simulations were performed with $n = 100$ neurons in order to ensure a sufficiently high level of angular precision, but in any case, the results are robust to the choice of ring size. As in ([2,3], Supplementary Figure 1), a rate model was adopted for the neurons in line with the following first-order ordinary differential equations:

$$\tau_E \frac{dc_i}{dt} = -c_i + g(\gamma_E + W^{I\rightarrow E}u + \sum_{i=1}^{n} W_{ji}^{E\rightarrow E} c_j + I_i) \tag{1}$$

where c_i is the activation of the i-th neuron in the ring, $g(x) = \max(0,x)$ is a rectified activation function,$\tau_E = 42.5$ ms is the time constant, $\gamma_E = -1.5$ is the activation threshold and I_i is the sensory input. The network comprises a single inhibitory neuron, which inhibits the neurons in the ring proportionally to its level of activation u with the weight $W^{I\rightarrow E} = -6$. Pairs of neurons (i, j) in the ring have symmetric excitatory connections and weights decreasing with the distance d_{ij} as follows:

$$W_{ji}^{E\rightarrow E} = \alpha \exp(-d_{ij}^2/2\sigma^2) \tag{2}$$

where $\alpha = 45/n$, $\sigma = 120°$ and $d_{ij} = |\theta_i - \theta_j|$. The temporal evolution of the inhibitory neuron is given by

$$\tau_I \frac{du}{dt} = -u + g(\gamma_I + W^{I\rightarrow I}u + W^{E\rightarrow I} \sum_{k=1}^{n} c_k) \tag{3}$$

$$\tag{4}$$

where $\tau_I = 2.125$ ms, γ_I=-7.5, $W^{I\rightarrow I} = -1$ and $W^{E\rightarrow I} = 60/n$ are the weight exerted by the inhibitory neuron on itself and that exerted by the whole population of excitatory neurons, respectively.

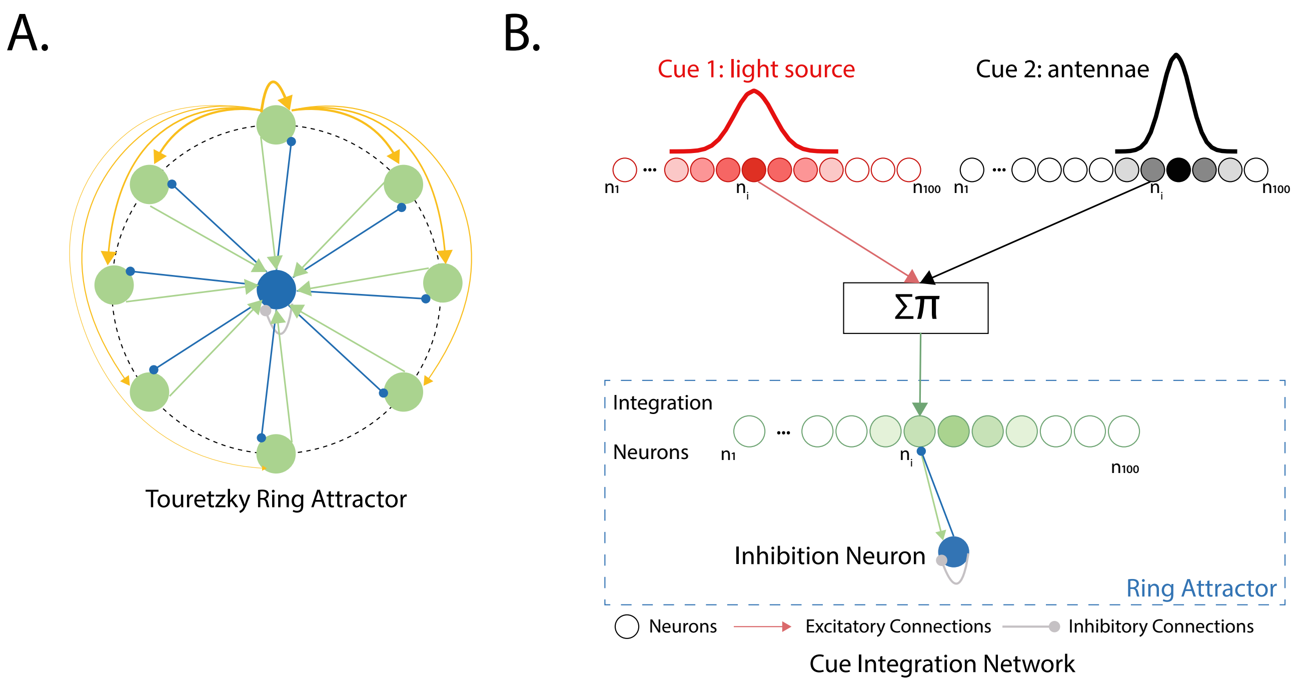

Supplementary Figure S 1. A and B adapted from[2], showing the Touretzky ring attractor network implemented here. A. Green circles stand for excitatory neurons, and the blue circle indicates the global inhibitory neuron. The orange arrows point to recurrent excitatory interneurons. The strength of the activation decreases with the distance between neurons. Excitatory and inhibitory connections between the global inhibitory neurons are shown in blue and green, respectively. B. The whole integration network is shown in unwrapped form (minus recurrent connections for the sake of simplification) with examples of inputs and optimal outputs superimposed. n = 100 neurons.

The model defined by Eq. (1) and (2) is known to give bell-shaped activation profiles[2,3]. The output of the ring is obtained over time as the winner-takes-all solution, that is, at each time step t, the winning neuron $i^{\star}(t)$ is that receiving the highest level of activation $c_{i^{\star}}(t) > c_i(t)\ \forall i$. Its preferred orientation gives the goal roll and its activation gives a scaling factor, computed as $K = 30\, c_{i^{\star}}(t)$, accounting for the roll dynamics.

Simulations were performed based on Euler integration, taking the step size $dt = 0.01$ ms. For the sake of simplicity, the inhibitory neuron was initialized at $u(t=0) = 1$, and the excitatory neurons were initialized with a bump at 0° corresponding to the proprioceptive cues sensed by the insect's legs prior to the experiments, that is $c_i(t=0) = W_{ji}^{E\to E}$ given by Eq. 2, where $j = 0$. During the fall, the leg proprioception was no longer taken into account as the legs were not in contact with a surface, whereas when $t > 0$, sensory cues of two other kinds, namely those provided by the visual system and the antennae, were taken into account. The visual input to neuron i was computed as follows:

$$X_{Vi} = \frac{k_V}{\sqrt{2\pi}\sigma_V} \exp(-|\theta_i - \mu_V|^2/2\sigma_V^2) \tag{5}$$

where $k_V = 40$, $\sigma_V = 10$ and $\mu_V = 0$ or 180 °, depending on whether the light originated from above or below, respectively. The antennal input to neuron i was computed as follows:

$$X_{Ai} = \frac{k_A}{\sqrt{2\pi}\sigma_A} \exp(-|\theta_i - \mu_A|^2/2\sigma_A^2) \tag{6}$$

where $\sigma_A = 5$, $\mu_A = 0$ ° and $k_A = 40$ or 0, depending on whether the antennae were intact or blocked with glue, respectively.

We first dealt with the classical linear neurons in the ring, to which the total input was the sum of the two sensory inputs, i.e. $I_i = X_{Ai} + X_{Vi}$. We did not completely succeed here, however, in simulating the experimental data because the difference in the roll rates observed between consistent and conflicting cues (the righting response was much faster with consistent cues) cannot be modeled by performing a simple summation of the sensory inputs, but requires some non-linear amplification. For this purpose, we used high-order, sigma-pi units[4,5] to which the total input was computed as follows:

$$I_i = X_{Ai} + X_{Vi} + \omega\, X_{Ai}\, X_{Vi} \tag{7}$$

where $\omega = 100$. When the two sensory cues disagree, the product $X_{Ai}\, X_{Vi} \approx 0$ and the sigma-pi unit behaves like a linear neuron with $I_i \approx X_{Ai} + X_{Vi}$, but when the two sensory cues are in agreement, a large product $X_{Ai}\, X_{Vi}$ is obtained, resulting in a non-linear amplification of the input I_i.

1.2 Closed-loop control of roll

We previously modeled the fly roll dynamics in the form of a purely second-order system (a double integrator, see Supplementary Figure 1 and[1]) receiving a torque U_{roll} as its input control signal and yielding a thorax roll speed Ω_{roll} and a thorax roll angle as its outputs, via the moment of inertia I_{roll}. As shown in Figure 1B, flies were able to reach the steady-state 0° position (right-side up) reliably. As described in Verbe et al., 2020[1], we modeled the exact closed-loop control of the roll by means of two nested feedback loops controlling the roll rate and the roll angle.

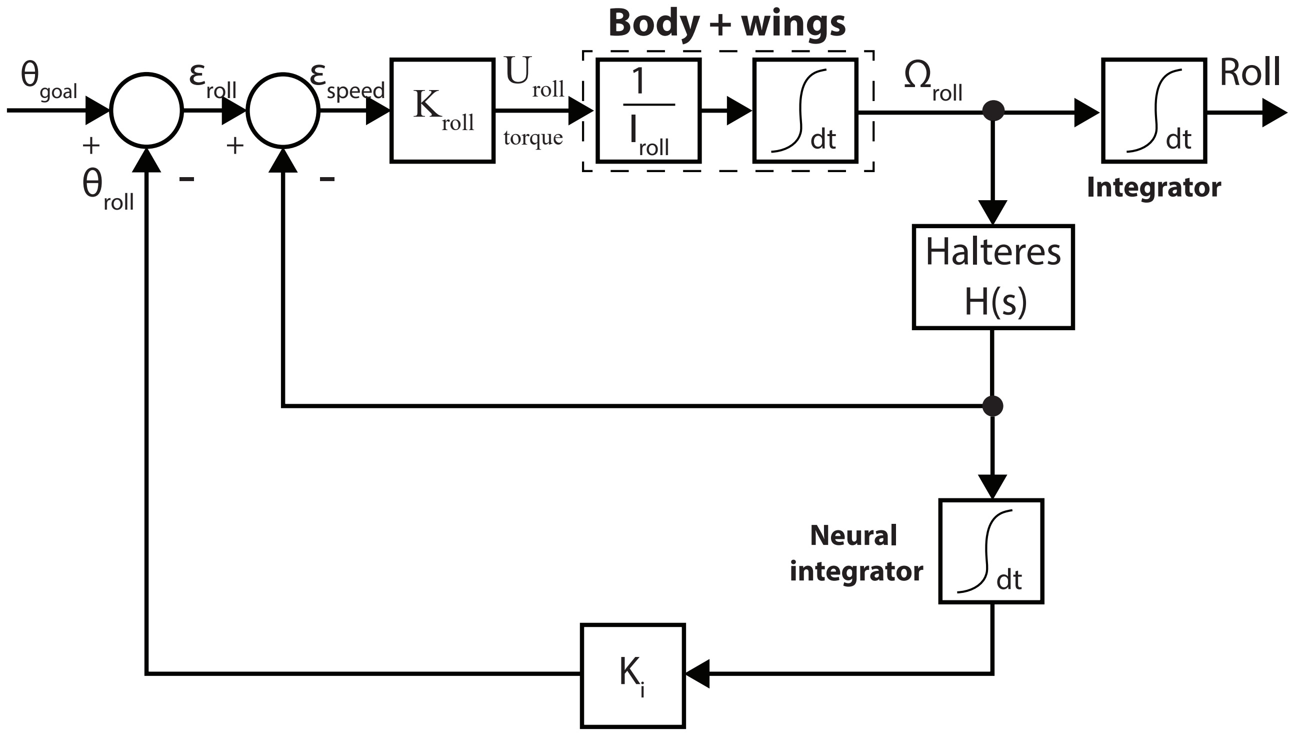

Supplementary Figure S 2. Dynamic model of hoverflies' righting reflex. Control block diagram including two nested feedback-loops: the fast feedback-loop controls the roll's angular speed measured by the halteres, and the slow loop controls the roll angle based on the estimated roll angle provided by a neural integrator. The goal roll signal (θ_{goal}) controls the amplitude of the body roll. ε_{roll} and ε_{speed} are error signals, K_{roll} is a gain, and U_{roll} is the torque roll. The multiplicative factor K_i (180/pi) serves merely to convert the estimated roll from radians to degrees. A simple integrator in the closed-loop mode (not shown here) is used to implement a low-pass filter with a variable time constant imposed by the K value. $K_{roll} = 1.22e-9$, $I_{roll} = 9.76\text{e-}12 kg.m^2$, $H(s) = \frac{1}{0.0035s+1}$.

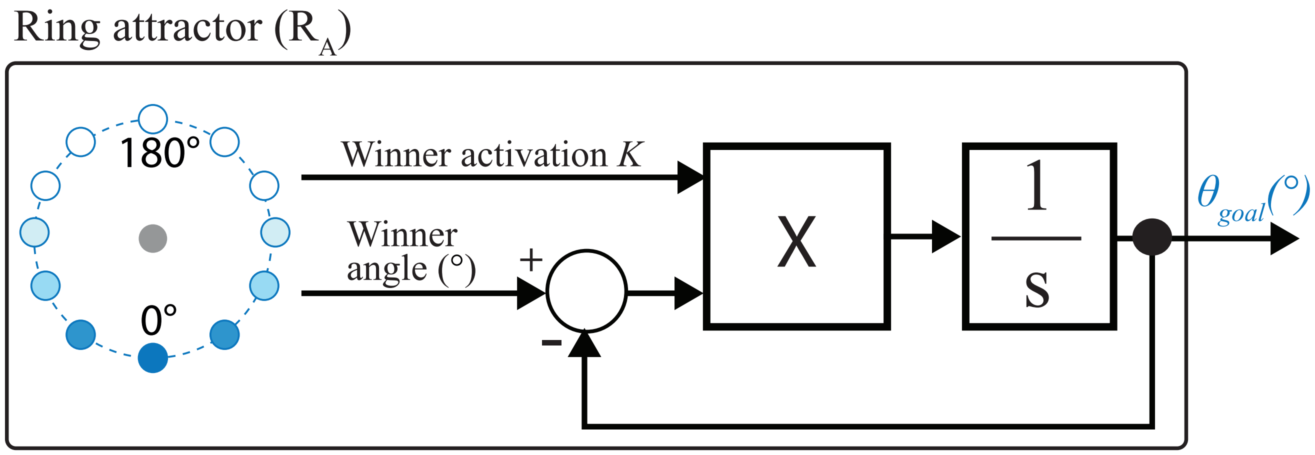

Supplementary Figure S 3. Implementation of the variable time constant low pass filter as classically done in analog computing[6]. A pure integrator ($1/s$ with s the Laplace variable) is placed in closed-loop to adapt the time constant by multiplying the error between the input signal (winner angle) and the output signal (θ_{goal}) with the winner activation K. As a result, the closed-loop transfer function corresponds to a first order low-pass filter with time constant $\tau_f = 1/K$.

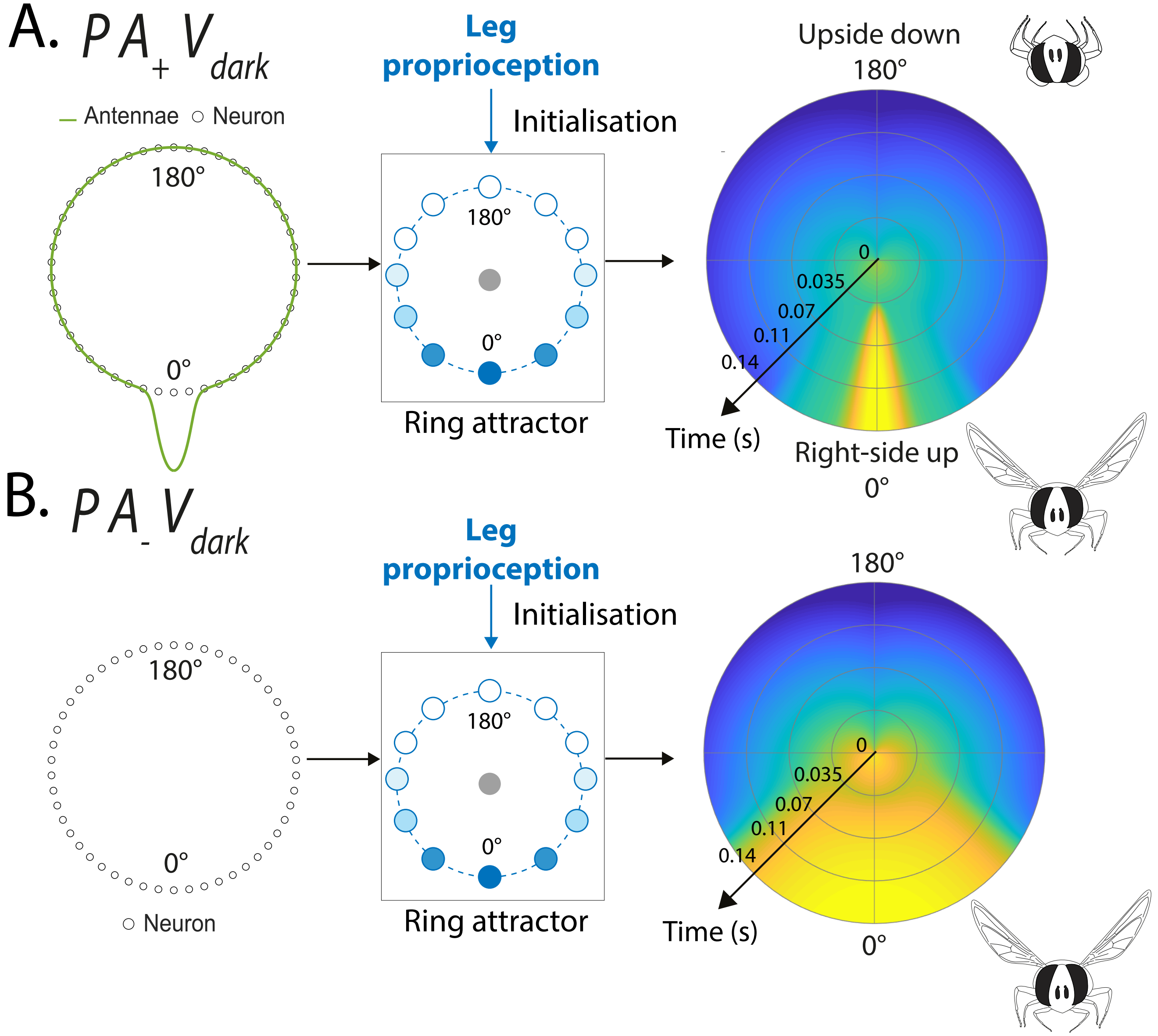

Supplementary Figure S 4. Ring attractor simulated responses in the two experimental conditions PA_+V_{dark} and PA_-V_{dark}. The output from the ring is obtained over time as the winner-take-all function, that is, at each time step, the winning neuron is that featuring the greatest activation. Winner angle codes for the goal roll value (Figure 2), whereas the value of K is equal to the amplitude of the winning neuron's activation. The initialization of the ring attractor is done by the leg's proprioceptive signal in the three conditions, and the integration of the orientation cues (Antenna and Light). In the case of PA_+V_{dark}, vision is absent and the antennae give the information corresponding to an upside-down position (0°), while in conditions PA_-V_{dark}, vision and the antennae are silent and only the leg proprioception generates information corresponding to an upside-down position (0°). The activation initiated at $t = 0$ is similar in both conditions: K = 0.45, winner angle = 0°. The final state at $t = 0.14$ s is K = 178, winner angle = 0° (panel A) and K=0.61, winner angle = 0° (panel B).

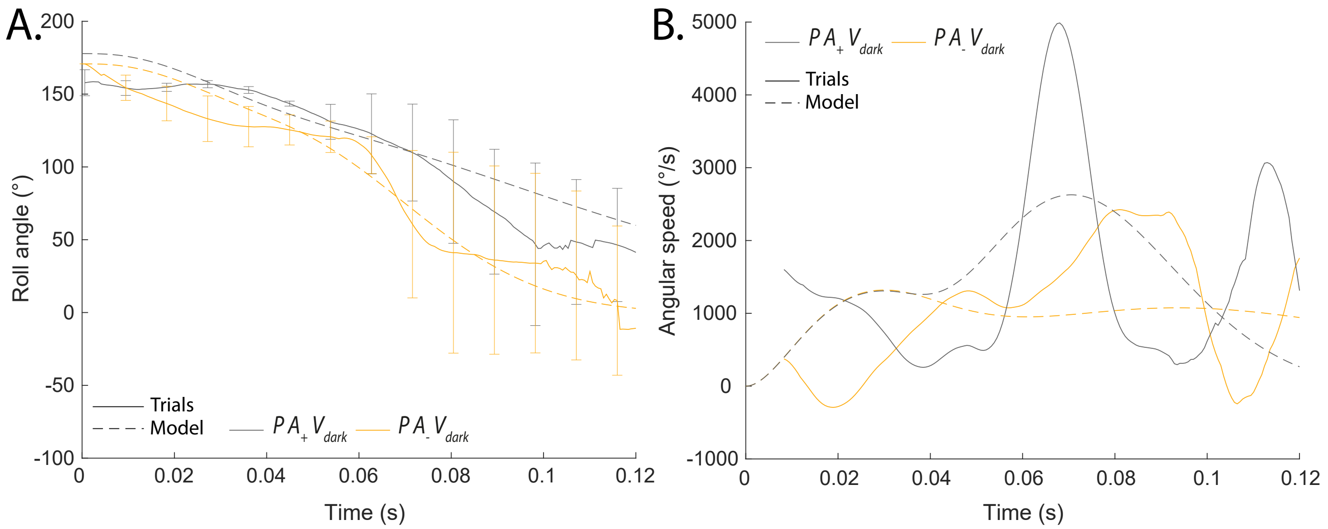

Supplementary Figure S 5. Response of the righting reflex model (dotted line) in comparison with the trials (solid line) in terms of the roll angle (A) and the angular speed (B) in the two dark conditions: with the antennae intact PA_+V_{dark} presented in gray and glued, PA_-V_{dark} in yellow. Thick lines are means, and error bars are S.Ds. See Supplementary Information, section 1.1 for further information.

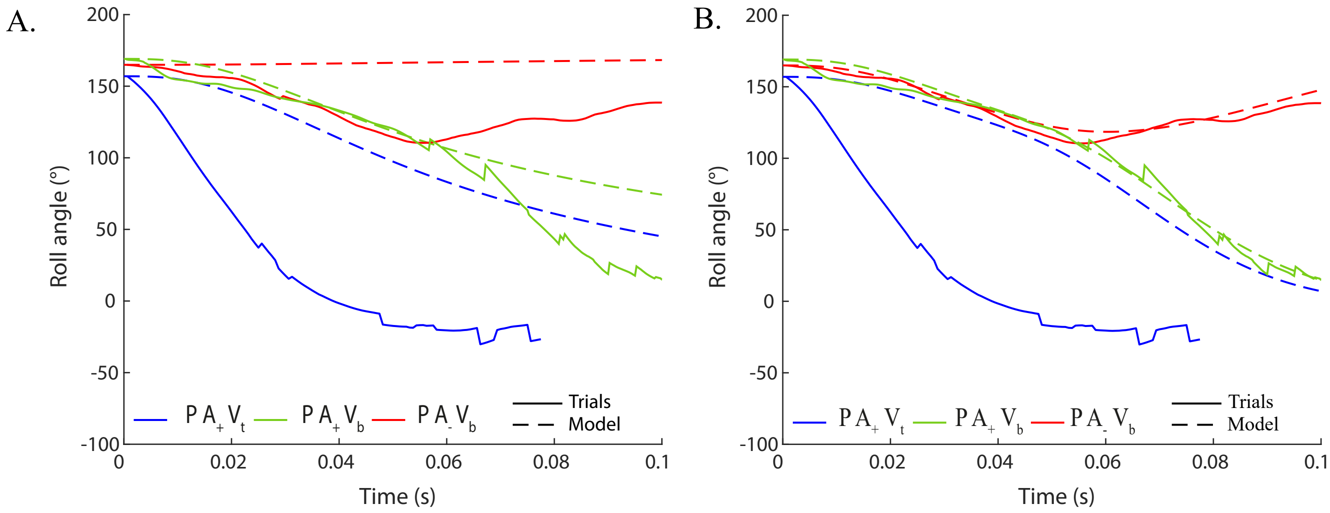

Supplementary Figure S 6. Linear models (dotted line) versus experimental data (solid line) in three different conditions. (A) Response of a linear integration model[7] that computes the winner angle in Supplementary Figure 2 as $\alpha\mu_A + (1-\alpha)\mu_V$ with $\alpha = \sigma_V^2/(\sigma_A^2+\sigma_V^2)$ (μ_A, μ_V and σ_A^2, σ_V^2 stand for the mean and variance of antennal and visual cues). The time constant of the output filter in Supplementary Figure 2 is here inversely proportional to the estimated variance given by $K = (\sigma_A\sigma_V)^2/(\sigma_A^2+\sigma_V^2)$. (B) Response of the ring attractor with purely linear neurons (in replacement of sigma-pi neurons), i.e. $\omega = 0$ in Eq. 1 in main text. The time constant of the output filter is inversely proportional to the winner activation as in Supplementary Figure 2.

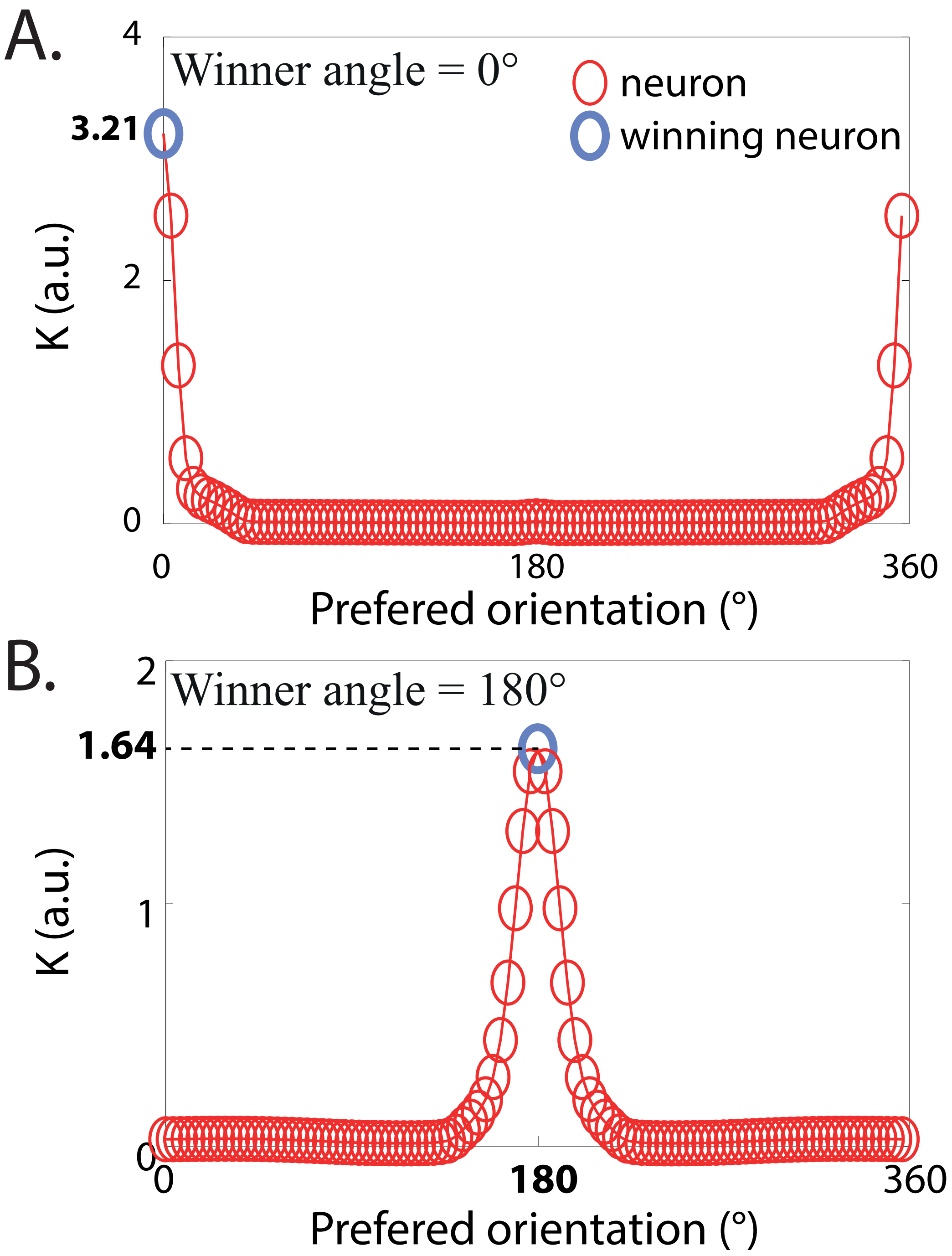

Supplementary Figure S 7. Activation profile of the ring attractor network at $t = 0.14$ s in two experimental conditions: $PA_{+}V_{b}$ in panel A and $PA_{-}V_{b}$ in panel B. Winner angle and K give the preferred roll orientation and the winning neuron's activation amplitude, respectively.

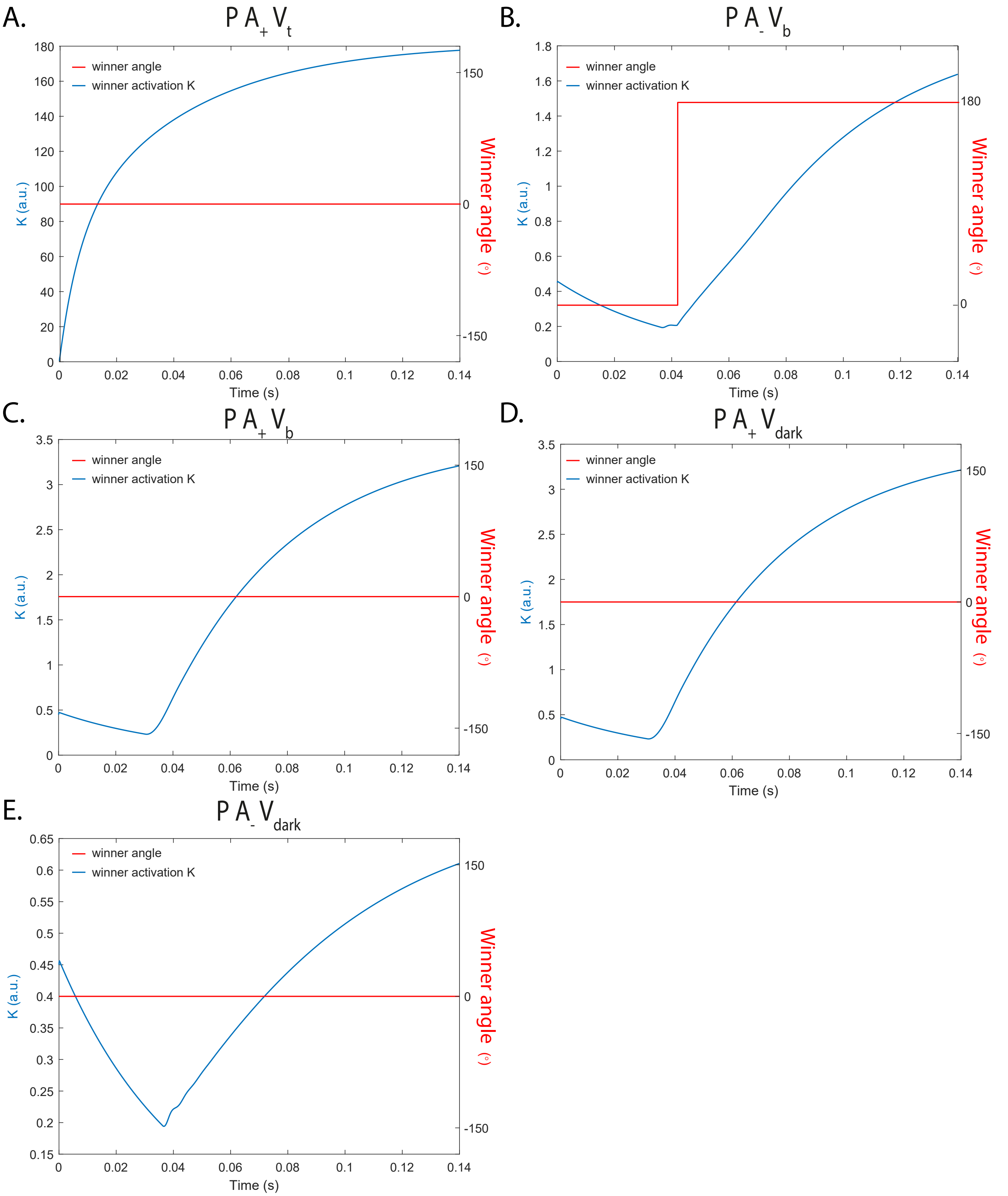

Supplementary Figure S 8. Time course of the winner angle and *K* (winner's preferred roll and activation amplitude) in the various experimental conditions.

Supplementary Movies

Supplementary Movie S1. Video of the hoverfly righting reflex and response of the model in the condition $PA_{+}V_{t}$.
Supplementary Movie S2. Video of the hoverfly righting reflex and response of the model in the condition $PA_{-}V_{b}$.
Supplementary Movie S3. Video of the hoverfly righting reflex and response of the model in the condition $PA_{+}V_{b}$.
Supplementary Movie S4. Video of the hoverfly righting reflex and response of the model in the condition $PA_{+}V_{dark}$.
Supplementary Movie S5. Video of the hoverfly righting reflex and response of the model in the condition $PA_{-}V_{dark}$.
Supplementary Movie S6. Video of the hoverfly righting reflex and response of the model in the condition $PA_{+}V_{t}$.

References

1. Verbe, A., Varennes, L. P., Vercher, J.-L. & Viollet, S. How do hoverflies use their righting reflex? *J. Exp. Biol.* **223**, DOI: 10.1242/jeb.215327 (2020). Publisher: The Company of Biologists Ltd Section: Research Article.

2. Sun, X., Mangan, M. & Yue, S. An analysis of a ring attractor model for cue integration. In *Conference on Biomimetic and Biohybrid Systems*, 459–470 (Springer, 2018).

3. Touretzky, D. S. Attractor network models of head direction cells. *Head direction cells neural mechanisms spatial orientation* 411–432 (2005). Publisher: MIT Press Cambridge, MA.

4. Durbin, R. & Rumelhart, D. E. Product Units: A Computationally Powerful and Biologically Plausible Extension to Backpropagation Networks. *Neural Comput.* **1**, 133–142, DOI: 10.1162/neco.1989.1.1.133 (1989).

5. Rumelhart, D. E., Hinton, G. E., McClelland, J. L. & others. A general framework for parallel distributed processing. *Parallel distributed processing: Explor. microstructure cognition* **1**, 26 (1986). Publisher: Cambridge, MA: MIT Press.

6. Ulmann, B. Analog computing. In *Analog Computing* (Oldenbourg Wissenschaftsverlag, 2013).

7. Ghahramani, Z., Wolpert, D. M. & Jordan, M. I. Computational models of sensorimotor integration. In Morasso, P. & Sanguineti, V. (eds.) *Advances in Psychology*, vol. 119 of *Self-Organization, Computational Maps, and Motor Control*, 117–147, DOI: 10.1016/S0166-4115(97)80006-4 (North-Holland, 1997).
